# Fluorescence Lifetime Imaging Microscopy of Porphyrins in *Helicobacter pylori* Biofilms

**DOI:** 10.3390/pharmaceutics13101674

**Published:** 2021-10-13

**Authors:** Antonella Battisti, Paola Morici, Antonella Sgarbossa

**Affiliations:** 1 Istituto Nanoscienze—CNR and NEST—Scuola Normale Superiore, Piazza S. Silvestro 12, I-56127 Pisa, Italy; antonella.battisti@nano.cnr.it (A.B.); paola.morici@hsanmartino.it (P.M.); 2 Ospedale Policlinico San Martino, Largo Rosanna Benzi 10, I-16132 Genova, Italy

**Keywords:** porphyrins, fluorescence lifetime imaging miscroscopy (FLIM), antimicrobial photodynamic therapy (aPDT), *Helicobacter pylori* biofilm

## Abstract

Bacterial biofilm constitutes a strong barrier against the penetration of drugs and against the action of the host immune system causing persistent infections hardly treatable by antibiotic therapy. *Helicobacter pylori* (Hp), the main causative agent for gastritis, peptic ulcer and gastric adenocarcinoma, can form a biofilm composed by an exopolysaccharide matrix layer covering the gastric surface where the bacterial cells become resistant and tolerant to the commonly used antibiotics clarithromycin, amoxicillin and metronidazole. Antimicrobial PhotoDynamic Therapy (aPDT) was proposed as an alternative treatment strategy for eradicating bacterial infections, particularly effective for Hp since this microorganism produces and stores up photosensitizing porphyrins. The knowledge of the photophysical characteristics of Hp porphyrins in their physiological biofilm microenvironment is crucial to implement and optimize the photodynamic treatment. Fluorescence lifetime imaging microscopy (FLIM) of intrinsic bacterial porphyrins was performed and data were analyzed by the ‘fit-free’ phasor approach in order to map the distribution of the different fluorescent species within Hp biofilm. Porphyrins inside bacteria were easily distinguished from those dispersed in the matrix suggesting FLIM-phasor technique as a sensitive and rapid tool to monitor the photosensitizer distribution inside bacterial biofilms and to better orientate the phototherapeutic strategy.

## 1. Introduction

Helicobacter pylori (Hp) can chronically colonize the human stomach where it plays an essential role in the development of gastritis, gastroduodenal ulcers and gastric cancer [1]. A key contribution to the persistence and the recurrence of Hp infections is provided by the bacterial ability to efficiently form a biofilm. Biofilms are matrix-enclosed bacterial populations in close proximity to each other and adherent to surfaces or interfaces. Hp biofilms are characterized by an extensive 3D network of highly hydrated exopolysaccharides where extracellular DNA, extracellular proteins, and outer membrane vesicles are embedded [2]. This extracellular matrix (ECM) protects the bacterial community from immune system cells and antimicrobial drugs. As a matter of fact, conventional pharmacological therapies have become less effective and new alternative strategies to fight bacterial infections are emerging. In this regard, one of the most promising approaches to overcome the antibiotic resistance problems is the photodynamic therapy (PDT). PDT combines the use of light irradiation with the presence of a photosensitizer molecule, able to generate cytotoxic reactive oxygen species (ROS) upon illumination. When this strategy is directed against microorganisms, the process is called antimicrobial photodynamic therapy (aPDT). The in situ-generated ROS are able to destroy biomacromolecules thus causing cell death. Typically, a photosensitizing drug is delivered to the area of interest before the treatment, but PDT can be particularly effective when the target microorganism naturally produces endogenous photosensitizers such as in the case of Hp porphyrins [3]. In previous studies we spectroscopically characterized the composition of Hp porphyrin extracts, showing that protoporphyrin IX (PPIX) and coproporphyrin I (CPI) are two of the main endogenous photosensitizing species [4,5]. Thanks to their presence, visible light irradiation conveyed by an intragastric fiberoptic laser system or by a LED-based illuminator has been shown to efficiently cause Hp photokilling without the administration of exogenous photosensitizers [6,7,8]. The correlation between photophysical properties and photosensitizing ability of porphyrins makes it mandatory to carefully analyze their fluorescence properties when they are closely associated with the biological target [9,10]. It has long been known that the vast majority of bacterial pathogens produce fluorescing porphyrins emitting in the red spectral region when excited by 405 nm violet light [11]. Thus, the assessment of red fluorescence in biological samples can be a signal of bacterial infection. Fluorescence imaging has recently emerged as a rapid and non-invasive technique for real-time visualization of the occurrence of bacteria and of their spatial distribution in several biological tissues and matrixes. The detection of the presence, location, and extent of fluorescent bacteria in wounds, for example, can be exploited by clinicians for targeted sampling during biopsies or to selectively treat the infected area [12,13]. Among the several fluorescence-based techniques, FLIM brings about several advantages because, in contrast to fluorescence intensity, the fluorescence decay time is independent of the local concentration of fluorophores and of the experimental set up. The fluorescence lifetime is a specific molecular feature of the fluorophore that can be sensitive to its surroundings. Fluorophores exhibiting the same spectral distribution in emission and in excitation as well may have different lifetimes, indicative of different molecular species or different conformations of the same molecule [14]. The analysis of FLIM data can be simplified by the phasor approach, a frequency domain technique that allows the transformation of the signal from every pixel in the image to a point in the phasor plot [15]. The FLIM-phasor approach has been used to create a metabolic fingerprint of individual bacteria and pop-ulations by monitoring the lifetime of the autofluorescent molecule reduced nicotinamide adenine dinucleotide phosphate (NAD(P)H). While the fluorescence spectra of free and bound NAD(P)H are very similar, their lifetimes differ significantly and depend on the metabolic state of bacteria. Phasor fingerprints were generated for *Lactobacillus acidophilus* [16], *Escherichia coli*, *Salmonella enterica* serovar Typhimurium, *Pseudomonas aeruginosa*, *Bacillus subtilis*, and *Staphylococcus epidermidis* [17] in different metabolic state during the growth phase or under antibiotic stress as well.

In this work, the phasor analysis coupled to FLIM was applied to Hp biofilms in order to map the distribution of the bacterial porphyrins in different microenvironments. Our findings show that it is possible to discriminate and image different average lifetimes for porphyrins corresponding to different molecular states inside bacteria or dispersed in the ECM. Therefore, this method can be used to provide a real-time assessment and a rapid visualization of the photosensitizer distribution along with its degree of molecular packing not only in Hp biofilms but also in all the situations where it is important to know the uptake, localization pattern and interactions of the exogenously added dye to optimize the PDT.

## 2. Materials and Methods

### 2.1. Bacterial Strains and Culture Conditions

Bacterial strains Hp ATCC^®^ 43504™ (laboratory-adapted) and Hp ATCC^®^ 700824™ (J99, virulent, cagA+ and vacA+), supplied by LGC Standards S.r.l. (Milan, Italy), were selected for this study. Both strains were kept at −80 °C in Brucella Broth (BB, Thermo Fisher Scientific Remel Products, Lenexa, KS, USA) supplemented with 20% (*v*/*v*) glycerol and 10% (*v*/*v*) heat-inactivated fetal bovine serum (FBS, Gibco, Life Technologies, Carlsbad, CA, USA). Thawed bacteria were grown in BB supplemented with 10% (*v*/*v*) FBS and incubated in the dark at 37 °C in a microaerophilic atmosphere (CampyGen Compact, Oxoid Hampshire, UK) under shaking (170 rpm).

### 2.2. Biofilm Formation Assay

Hp strains from frozen stocks were cultured on Brucella agar plates supplemented with 7% laked horse blood (Oxoid, Hampshire, UK) and incubated for 3 days at 37 °C under microaerophilic conditions in accordance with previously published protocols [18]. A sterile cotton swab was used to harvest bacteria after incubation. Bacteria were then suspended in brain heart infusion (BHI) broth (Oxoid, Hampshire, UK) additioned with 0.5% β-cyclodextrin (Sigma-Aldrich, St. Louis, MO, USA) and 0.4% yeast extract (Oxoid, Hampshire, UK). Aliquots of 2 mL of this suspension, adjusted to 5 × 10^6^ CFU/mL concentration, were inoculated in the wells of 12-well culture plates (BD Falcon, Franklin Lakes, NJ, USA), where sterilized round glass coverslips were placed vertically in order to promote the adhesion of Hp cells at the air-liquid interface. BHI broth (without bacteria) was the negative control. Plates were incubated for 4 days at 37 °C under microaerophilic conditions. Biofilms grown on the glass coverslips were washed twice with phosphate-buffered saline (PBS, Gibco, Life Technologies, Carlsbad, CA, USA) to remove excess bacterial cells and immediately set for imaging at room temperature.

### 2.3. Extraction of Porphyrins from Hp Strains

According to a previously published protocol [19] with minor modifications, Hp cells of different ages (1–4 days) were centrifugated at 4 °C (7000× *g* for 10 min), washed in 20 mL pre-cooled buffer (0.05 M Tris, 2 mM EDTA, pH 8.2) and resuspended in 10 mL of the same buffer. An aliquot of 1.5 mL of a mixture of ethyl acetate and acetic acid (3:1, *v*/*v*) was added to the suspension and bacterial cells were lysed by sonication in ice (6 cycles as in the following: 30 s, stop, 60 s). Bacterial debris was removed by centrifugation at 4 °C (7000× *g* for 10 min), and the organic layer was washed twice with distilled water. Por-phyrins were extracted from the organic phase by adding 150 μL of HCl 3M. After fast vortexing, this solution was centrifuged for 5 min at 7000× *g* and then the bottom aqueous layer was collected for chromatographic analyses.

### 2.4. High Performance Liquid Chromatography

The HPLC used for analyses was a Dionex Ultimate 3000 HPLC (ThermoFisher Scientific Inc., Waltham, MA, USA), equipped with a fraction collector, PDA detector and a (3 × 150 mm) Kinetex PFP column (Phenomenex Inc., Torrance, CA, USA) using water/formic acid 100/0.1 *v*/*v* (A) and acetonitrile/formic acid 100/0.1 *v*/*v* (B) as mobile phases, with a flow of 0.8 mL/min. Runs were performed in four steps as follows: 2 min at 25% B, 26 min with a linear gradient up to 95% B, 4 min purge step at 95% B and 8 min re-equilibration step.

### 2.5. Fluorescence Lifetime Imaging (FLIM)

Standard porphyrins PPIX and CPI were either analyzed in methanol solution (about 1 × 10^−5^ M) or crystallized from solution on a glass coverslip by slow evaporation of the solvent. The measurements were performed using a Leica TCS SP5 inverted confocal microscope (Leica Microsystems, Wetzlar, Germany). An external pulsed diode laser provided excitation at 405 nm and 640 nm, while a TCSPC acquisition card (PicoHarp 300, PicoQuant, Berlin, Germany) connected to internal spectral photomultipliers allowed for detection. On the basis on the lifetime values, laser repetition rate was fixed at 20 or 40 Hz. Image size was set to 256 × 256 pixels and scan speed was modulated between 200 and 400 Hz (lines per second). The detection wavelength range was set between 580 and 720 nm for λ_ex_= 405 nm and between 650 and 750 nm for λ_ex_= 640 nm thanks to the built-in AOBS detection system. About 200–300 photons per pixel were collected for each measurement, at a photon counting rate of 100–200 kHz. Data collected from different ROIs in the biofilms images were averaged. Globals for Images—SimFCS v.2 (Globals Software by LFD-UCI, Irvine, CA, USA, available at www.lfd.uci.edu, accessed on 1 September 2021) was used to calculate phasors from the FLIM images.

## 3. Results

### 3.1. Chromatographic Analysis of Hp Porphyrin Production

**Figure 1:**
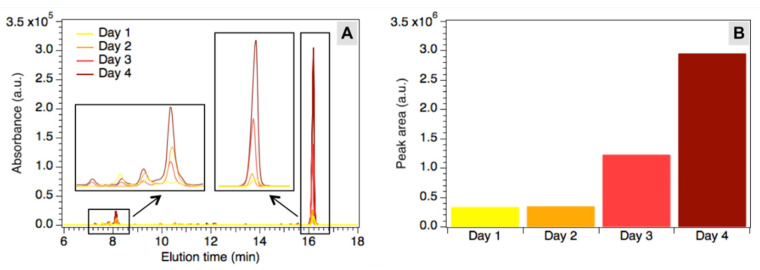
(**A**) Chromatograms of the extracts after 1 up to 4 days. Insets: expansion of peaks between 7.0 and 8.5 min and between 15.7 and 16.7 min (rescaled for better visualization). (**B**) Total area under the main peaks in the chromatograms (7.0–8.5 min and 15.7–16.7 min), corresponding to the global concentration of fluorescent species in the extracts.

Bacterial extracts were obtained from Hp cultures of different ages (1–4 days) by means of a specific protocol and characterized by HPLC analyses as described in Materials and Methods. The main peaks in the chromatograms were attributed to fluorescent porphyrins and flavins produced by bacteria, according to previously published results [5,6]. HPLC allowed for a comparison between the concentrations of the fluorescent species at different ages of the bacterial cultures in order to follow the production of these molecules with time (Figure 1A). As shown by the increasing height of the peaks, the fluorophores accumulate during the observed time span, suggesting a continuous production and accumulation. To visualize changes in the global fluorophores concentration, the main known peaks in the chromatograms (7.0–8.5 min and 15.7–16.7 min) were integrated and the total calculated area was reported in Figure 1B as a function of culture age. After a 2-day lag phase, where no significant change can be noticed, the global concentration of fluorophores starts to grow reaching almost a tenfold increase after four days.

### 3.2. FLIM-Phasor Analysis of Porphyrins in Hp Biofilms

**Figure 2:**
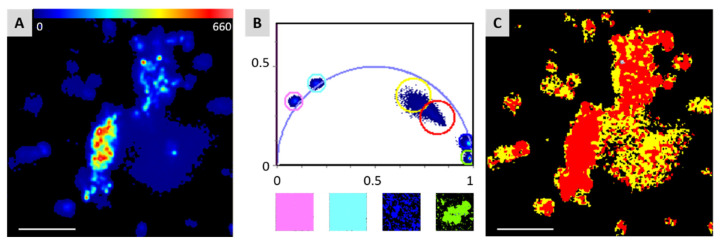
(**A**) False-color intensity-based image of bacterial biofilm. (**B** top) Overlap of the phasor plots of standard porphyrins PPIX (pink circle: methanol solution, green circle: crystal) and CPI (cyan circle: methanol solution, blue circle: crystal) and biofilm sample (red circle: bacteria, yellow circle: biofilm matrix). (**B** bottom) Phasor images of the standards, color code as in the plot. (**C**) Corresponding phasor image of the biofilm in A. Scale bar in A and C: 25 μm.

Fluorescence microscopy of Hp biofilms revealed that bacterial porphyrins are present both inside bacterial cells and in the ECM [4], as they confer red fluorescence to the whole system. Biofilms obtained from Hp after four days of incubation were then observed by confocal laser scanning microscopy and analyzed by the FLIM-phasor technique developed by Jameson et al. [15]. Figure 2A shows a false-color image of a fluorescent Hp biofilm where bacteria can be easily distinguished from the matrix thanks to the higher fluorescence intensity. Conversion of the FLIM data to the phasor plot produced a cloud of pixels inside the universal circle (Figure 2B), revealing a multiexponential fluorescence lifetime decay. The corresponding phasor image in Figure 2C clearly shows that different areas of the biofilm (bacteria/ECM) are associated with different positions in the phasor plot. To inspect the behavior of porphyrins lifetime in different contexts, PPIX and CPI were also analyzed as monomers dissolved in methanol solutions and in their crystalline form. All these samples gave strictly monoexponential decays, which fall on the edge of the universal circle in the phasor plot (Figure 2B). As expected, crystalline porphyrins gave pixels clouds falling in the bottom-right area of the plot, corresponding to very short lifetimes values (<1 ns), while monomeric porphyrins in methanol solution produced pixels in the left part of the phasor plot, with lifetimes exceeding 10 ns.

## 4. Discussion

Bacterial biofilms are composed of living cells embedded in an extracellular polymeric matrix along with several exocellular molecules excreted by the cells or captured from the environment. When bacterial cells die, they release substances in the ECM, mainly DNA along with other endogenous cytoplasmic components. Dead cells can be a supplier for the ECM because the leakage of cytoplasmic components due to bacterial cell lysis has been shown to favor biofilm formation [20]. On this basis, it is not surprising to find endogenous Hp porphyrins both inside the bacterial cells and dispersed in the ECM of Hp biofilms.

The chromatographic analysis performed on Hp extracts after different periods of culture revealed that porphyrins are produced from the first day, but their concentration drastically increases after 4 days of incubation. Although the planktonic and biofilm growing conditions are rather different, after 4 days fully formed biofilms likewise provided a sufficient concentration of porphyrins for analysis. Accordingly, Hp biofilms were grown for 4 days and analyzed by FLIM coupled to the phasor approach.

It is known that intracellular lifetime measurements on porphyrins usually produce three lifetime values, generally attributed to the monomeric (>10 ns), dimeric (1.5–3.0 ns) and highly aggregated (<1 ns) forms [21], but these Hp biofilms do not show lifetimes >10 ns, as if no porphyrin monomers were present [4]. Nevertheless, the positioning of pixels inside the universal circle in the phasor plot matches with the presence of a combination of different lifetimes (Figure 2B). Monoexponential decays fall on the edge of the universal circle, as in the case of monomeric (pink and cyan circles) or crystalline (blue and green circles) porphyrins. The area where the biofilm pixels fall suggests that porphyrins are mostly present in several aggregated forms, likely in a mixture of dimers and higher oligomers, whose lifetimes are combined to produce the observed pixel cloud. Notably, the phasor analysis applied to FLIM acquisitions of Hp biofilms reveals different average lifetimes for porphyrins inside bacteria or dispersed in the ECM, as expressed by the slightly different position of the red and yellow circles in the plot of Figure 2B, corresponding to the red (bacteria) and yellow (matrix) areas in the phasor image (Figure 2C). This could be attributed either to a different composition between the intracellular and extracellular porphyrins mixture or to their different aggregation grade inside and outside the cells, along with the different environmental interactions. It is worth noting how the phasor approach allows for easy discrimination of the two different fluorescent areas.

## 5. Conclusions

To the best of our knowledge, in this work the FLIM-phasor approach was employed for the first time to easily discriminate porphyrins located in different fluorescent areas of Hp biofilms. This could be exploited to assess the extent of bacterial contamination and the presence of a biofilm inside an infected tissue, suggestive of an advanced stage of infection, thus simplifying the choice of the best treatment. Our findings indicate that porphyrins inside the cells and dispersed in the ECM show a slightly different global lifetime; likely, on the basis of the position of the relevant pixels cloud in the phasor plot, intracellular porphyrins may be organized in more packed structures. From the phototherapeutical point of view, this knowledge can be of great importance because, due to the short diffusion path of singlet oxygen and other cytotoxic reactive oxygen species, the more strictly the photosensitizer is bound to the cellular target, the more effective its photokilling activity will be. In addition, as the intracellular pigment concentration varies with the age of bacteria, the detection and the spatial distribution pattern of porphyrin fluorescence in Hp biofilm could provide some hints for the choice of the most appropriate therapeutical time window to perform aPDT. In general, this kind of analysis could be considered to be a useful and rapid tool for the exploration of fluorescent bacterial biofilms properties not only to obtain new insights into the biofilm structure and dynamics but also to develop and optimize novel antimicrobial strategies.

## Data Availability

Data are available from the authors upon request.

## References

[1] Burkitt M.D., Duckworth C.A., Williams J.M., Pritchard D.M. (2017). *Helicobacter pylori*-induced gastric pathology: Insights from in vivo and ex vivo models. Dis. Model. Mech..

[2] Krzyżek P., Grande R., Migdał P., Paluch E., Gościniak G. (2020). Biofilm Formation as a Complex Result of Virulence and Adaptive Responses of *Helicobacter pylori*. Pathogens.

[3] Hamblin M.R., Viveiros J., Yang C., Ahmadi A., Ganz R.A., Tolkoff M.J. (2005). *Helicobacter pylori* accumulates photoactive porphyrins and is killed by visible light. Antimicrob. Agents Chemother..

[4] Battisti A., Morici P., Ghetti F., Sgarbossa A. (2017). Spectroscopic characterization and fluorescence imaging of *Helicobacter pylori* endogenous porphyrins. Biophys. Chem..

[5] Battisti A., Morici P., Signore G., Ghetti F., Sgarbossa A. (2017). Compositional analysis of endogenous porphyrins from *Helicobacter pylori*. Biophys. Chem..

[6] Morici P., Battisti A., Tortora G., Menciassi A., Checcucci G., Ghetti F., Sgarbossa A. (2020). The in vitro photoinactivation of *Helicobacter pylori* by a novel LED-based device. Front. Microbiol..

[7] Ganz R.A., Viveiros J., Ahmad A., Ahmadi A., Khalil A., Tolkoff M.J., Nishioka N.S., Hamblin M.R. (2005). *Helicobacter pylori* in patients can be killed by visible light. Lasers Surg. Med..

[8] Lembo A.J., Ganz R.A., Sheth S., Cave D., Kelly C., Levin P., Kazlas P.T., Baldwin P.C., Lindmark W.R., Hamlin M.R. (2009). Treatment of *Helicobacter pylori* infection with intra-gastric violet light phototherapy: A pilot clinical trial. Lasers Surg. Med..

[9] Rennie M.Y., Dunham D., Lindvere-Teene L., Raizman R., Hill R., Linden R. (2019). Understanding Real-Time Fluorescence Signals from Bacteria and Wound Tissues Observed with the MolecuLight i:XTM. Diagnostics.

[10] Blackshaw E.L., Jeffery S.L.A. (2018). Efficacy of an imaging device at identifying the presence of bacteria in wounds at a plastic surgery outpatients clinic. J. Wound Care.

[11] Philipp-Dormston W.K., Doss M. (1973). Comparison of porphyrin and heme biosynthesis in various heterotrophic bacteria. Enzyme.

[12] Lopez A.J., Jones L.M., Reynolds L., Diaz R.C., George I.K., Little W., Fleming D., D’Souza A., Rennie M.Y., Rumbaugh K.P. (2021). Detection of bacterial fluorescence from in vivo wound biofilms using a point-of-care fluorescence imaging device. Int. Wound J..

[13] Jones L.M., Dunham D., Rennie M.Y., Kirman J., Lopez A.J., Keim K.C., Little W., Gomez A., Bourke J., Ng H. (2020). In vitro detection of porphyrin-producing wound bacteria with real-time fluorescence imaging. Future Microbiol..

[14] Suhling K., French P.M.W., Phillips D. (2005). Time-resolved fluorescence microscopy. Photochem. Photobiol. Sci..

[15] Jameson D.M., Gratton E., Hall R.D. (1984). The measurement and analysis of heterogeneous emissions by multifrequency phase and modulation fluorometry. Appl. Spectrosc. Rev..

[16] Torno K., Wright B.K., Jones M.R., Digman M.A., Gratton E., Phillips M. (2013). Real-time Analysis of metabolic activity within *Lactobacillus acidophilus* by phasor fluorescence lifetime imaging microscopy of NADH. Curr. Microbiol..

[17] Bhattacharjee A., Datta R., Gratton E., Hochbaum A.I. (2017). Metabolic fingerprinting of bacteria by fluorescence lifetime imaging microscopy. Sci. Rep..

[18] Yang F.L., Hassanbhai A.M., Chen H.Y., Huang Z.Y., Lin T.L., Wu S.H., Ho B. (2011). Proteomannans in biofilm of *Helicobacter pylori* ATCC 43504. Helicobacter.

[19] Mancini S., Imlay J.A. (2015). Bacterial porphyrin extraction and quantification by LC/MS/MS analysis. Bio-protocol.

[20] Flemming H.C., Wingender J. (2010). The biofilm matrix. Nat. Rev. Microbiol..

[21] Ricchelli F. (1995). Photophysical properties of porphyrins in biological membranes. J. Photochem. Photobiol. B Biol..

